# The British Sleep Society position statement on Daylight Saving Time in the UK

**DOI:** 10.1111/jsr.14352

**Published:** 2024-10-23

**Authors:** Megan R. Crawford, Eva C. Winnebeck, Malcolm von Schantz, Maria Gardani, Michelle A. Miller, Victoria Revell, Alanna Hare, Caroline L. Horton, Simon Durrant, Joerg Steier

**Affiliations:** ^1^ University of Strathclyde Centre for Sleep Health, Department of Psychological Sciences and Health University of Strathclyde Glasgow UK; ^2^ Section of Chronobiology, School of Biosciences, Faculty of Health and Medical Sciences University of Surrey Guildford UK; ^3^ Faculty of Health and Life Sciences Northumbria University Newcastle‐upon‐Tyne UK; ^4^ School of Health in Social Science, Department of Clinical and Health Psychology University of Edinburgh Edinburgh UK; ^5^ Directorate of Warwick Applied Health, Warwick Medical School University of Warwick Coventry UK; ^6^ Surrey Sleep Research Centre, Faculty of Health and Medical Sciences University of Surrey Guildford UK; ^7^ Department of Sleep and Ventilation Royal Brompton and Harefield Hospitals London UK; ^8^ Honorary Clinical Senior Lecturer, NHLI Imperial College London London UK; ^9^ Department of Psychology Bishop Grosseteste University Lincoln UK; ^10^ Lincoln Sleep Research Centre University of Lincoln Lincoln UK; ^11^ School of Psychology University of Lincoln Lincoln UK; ^12^ Centre for Human and Applied Physiological Sciences Faculty of Life Sciences and Medicine, King's College London London UK

**Keywords:** circadian clock, circadian timing systems, clock change, clock time, Daylight Saving Time, sleep, Standard Time

## Abstract

There is an ongoing debate in the United Kingdom and in other countries about whether twice‐yearly changes into and out of Daylight Saving Time should be abolished. Opinions are divided about whether any abolition of Daylight Saving Time should result in permanent Standard Time, or year‐long Daylight Saving Time. The British Sleep Society concludes from the available scientific evidence that circadian and sleep health are affected negatively by enforced changes of clock time (especially in a forward direction) and positively by the availability of natural daylight during the morning. Thus, our recommendation is that the United Kingdom should abolish the twice‐yearly clock change and reinstate Standard Time throughout the year.

## 1 BACKGROUND AND HISTORICAL OVERVIEW OF DAYLIGHT SAVING TIME (DST) IN THE UK

Daylight Saving Time (DST), or British Summer Time as it is widely referred to in the UK, consists of moving our clocks forward by 1 hour on the last Sunday in March and then back again to Standard Time on the last Sunday in October. Prior to the implementation of DST, the UK lived by Standard Time (equivalent to Greenwich Mean Time, GMT) for the whole year. Standard Time aligns closely with the natural light–dark cycles of day and night (solar time). This means that at Greenwich, the sun is at the highest point in the sky (solar noon) at midday GMT (noon time). By comparison, DST at Greenwich is always an hour ahead of solar time. DST was first temporarily introduced in the UK during the First World War after it was implemented by Germany and its allies. During the Second World War, the UK was even on double DST (2 hours ahead of Standard Time). The current arrangement of a regular twice‐yearly switch of the clocks began in 1972 with the British Summer Time Act.

In 2018, the European Parliament voted to ask the European Commission to re‐evaluate the future of DST in Europe (European Commission, 2018). In an online poll, respondents voted in favour of the abolition of twice‐yearly clock changes. In the UK, no progress has been made either to act (or not) on this European vote (much like in the other countries that have remained in the EU). There remains intense discussion around abolishing the clock change. Proposals include not only restoring Standard Time all year around, but also an alternative option of implementing permanent DST in the UK. In this position statement, we present why, from a sleep and circadian health perspective, permanent Standard Time (and **not** permanent DST) is recommended.

## 2 THE EFFECTS OF LIGHT EXPOSURE ON OUR CIRCADIAN RHYTHMS, AND HOW THIS COULD BE AFFECTED BY PERMANENT DST

Our body clocks, which drive our circadian rhythms (*circa diem* = approx. a day, thus any rhythms that have ~24‐hr cycles), are essential for the correct daily timing of our bodily functions (e.g. sleep/wake rhythms, gene expression, hormones, metabolism, mood; Czeisler et al., 1986). What keeps them aligned to the 24‐hr day is adequately timed light exposure (Czeisler et al., 1999). During the early hours of the morning, light exposure brings clocks and rhythms forward, while during the evening, it delays them (Roenneberg & Foster, 1997). Humans have a natural tendency to delay (Roenneberg & Foster, 1997), which is exacerbated by our modern lifestyle where we spend most of our time indoors and use artificial light in the evening, which tends to delay circadian rhythms. Therefore, morning light plays a central role in preventing our body clocks from becoming too late and in aligning them adequately with the 24‐hr day.

This also means that morning light is crucial to allow us to initiate sleep early enough in the evening, and wake early enough in the morning (preferably naturally without an alarm clock) for work and school starts. By contrast, light exposure during the late evening delays sleep onset and our natural waking, making it difficult to get up in the morning (which is then still our body's nighttime given the circadian delay) and to obtain sufficient amounts of sleep. In particular, individuals with very early work schedules or those struggling with too late sleep times for their schedules, such as teenagers who are prone to circadian delays, require optimal light exposure patterns to maintain adequate sleep patterns and durations. Although both sunlight and electric light exert these effects on our body clocks, sunlight has much greater potency in this circadian synchronisation because of its brightness and spectral quality (Brown et al., 2022). Disruption of the daily synchronisation of our body clocks causes disturbances in our physiology and behaviour including sleep (as shown in laboratory studies; Buxton et al., 2012; Wu et al., 2012) which leads to negative short‐ and long‐term physical and mental health outcomes (as shown by epidemiological studies; Burns et al., 2021; Cho, 2001; Daghlas et al., 2021; Jones et al., 2019; Roenneberg et al., 2012; Vetter et al., 2016).

The custom of switching our clocks twice‐yearly impacts our behaviour in relation to the timing of the opportunity for sunlight exposure. It is sometimes erroneously assumed that DST provides us with more sunlight but, in fact, all we are doing is changing our behaviour by moving our schedules forward by 1 hr. While this means there is an hour more sunlight *after* work/school, DST comes at an expense of 1 hr less sunlight *before* work/school, simply because we get up and travel to and from work/school 1 hr earlier. During summer, sunrise in the UK is early enough, so that for most of the population the reduced opportunity for morning light is only theoretical. This is because most people's natural wake time is much later than sunrise during our summer months (Table 1, column 7). However, the possibility of permanent DST (beyond summer and into winter) poses a potential danger to human sleep and health, primarily because of the resulting lack of natural light during winter mornings (Table 1, column 9). Sunrises in winter occur considerably later than in summer, so if we were to get up an hour earlier as DST demands, this would result in a lack of natural light in the morning before we start our day. This would reduce our opportunity to advance our body clocks and obtain adequate sleep. Several position statements, all of them arguing against the implementation of permanent DST, have been published by international sleep and biological clocks learned societies (Malow, 2022; Rishi et al., 2024; Roenneberg et al., 2019). However, none of these address the UK perspective specifically, and the impact that permanent DST would likely have on the UK population. To do this, we need to consider the unique geographical context of the UK.

**TABLE 1:** Overview of sunrise times in the scenario of permanent British Standard Time (in 2024) and permanent DST with data sourced from https://www.timeanddate.com/.

	Permanent Standard Time option				Permanent DST time option			
	Sunrise				Sunrise			
	Spring equinox	Summer solstice	Autumn equinox	Winter solstice	Spring equinox	Summer solstice	Autumn equinox	Winter solstice
City	Time (hours)				Time (hours)			
London	06:02	03:43	05:47	08:03	07:02	04:43	06:47	09:03
Birmingham	06:09	03:44	05:54	08:16	07:09	04:44	06:54	09:16
Swansea	06:17	03:57	06:02	08:19	07:17	04:57	07:02	09:19
Glasgow	06:18	03:31	06:03	08:45	07:18	04:31	07:03	09:45
Derry /Londonderry	06:30	03:49	06:15	08:52	07:30	04:49	07:15	09:52

DST, Daylight Saving Time.

## 3 THE GEOGRAPHICAL CONTEXT

**FIGURE 1:**
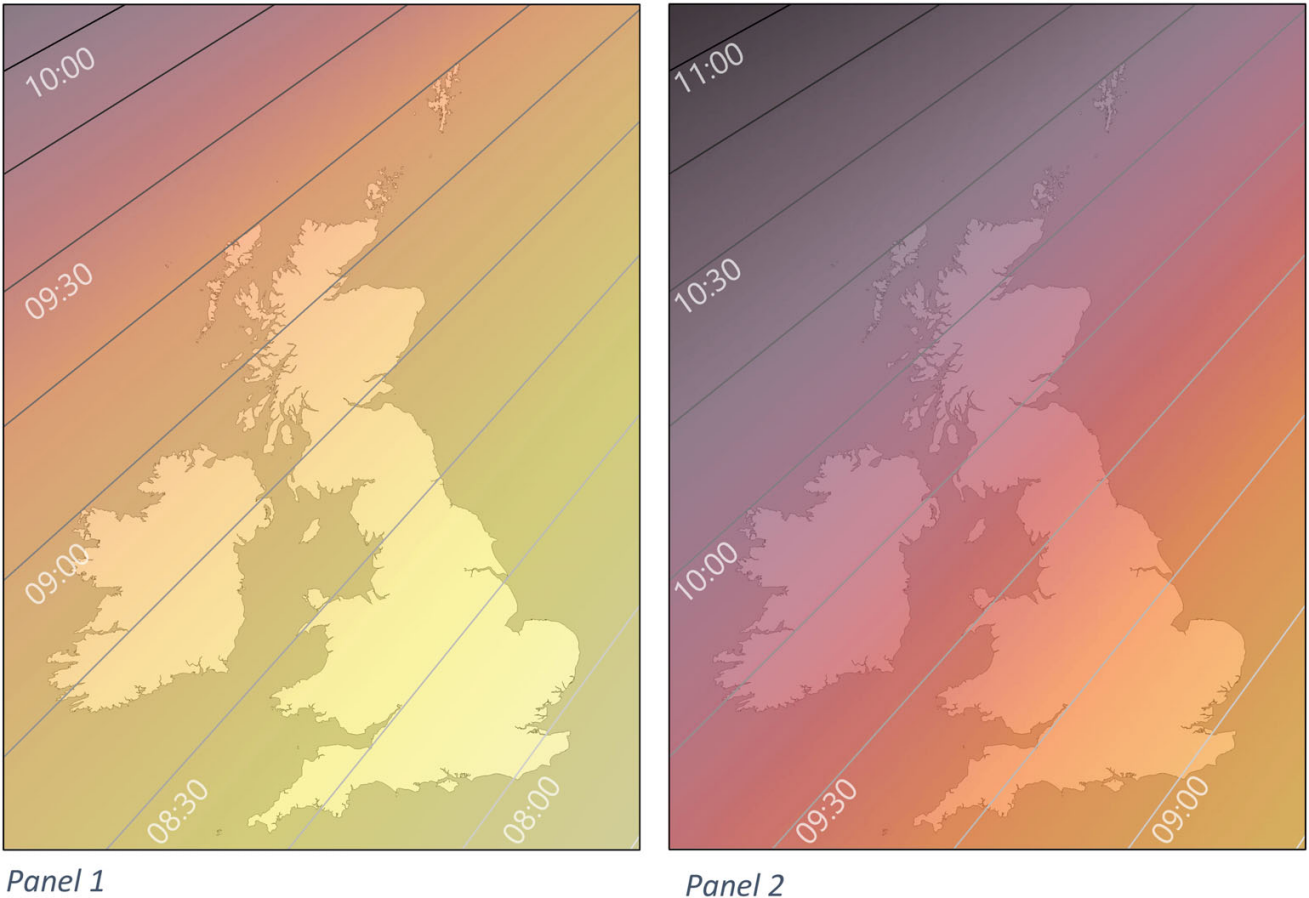
Sunrise times across the UK on winter solstice. Panel 1 illustrates sunrise time during permanent Standard Time. Panel 2 illustrates sunrise time during permanent Daylight Saving Time (DST).

**FIGURE 2:**
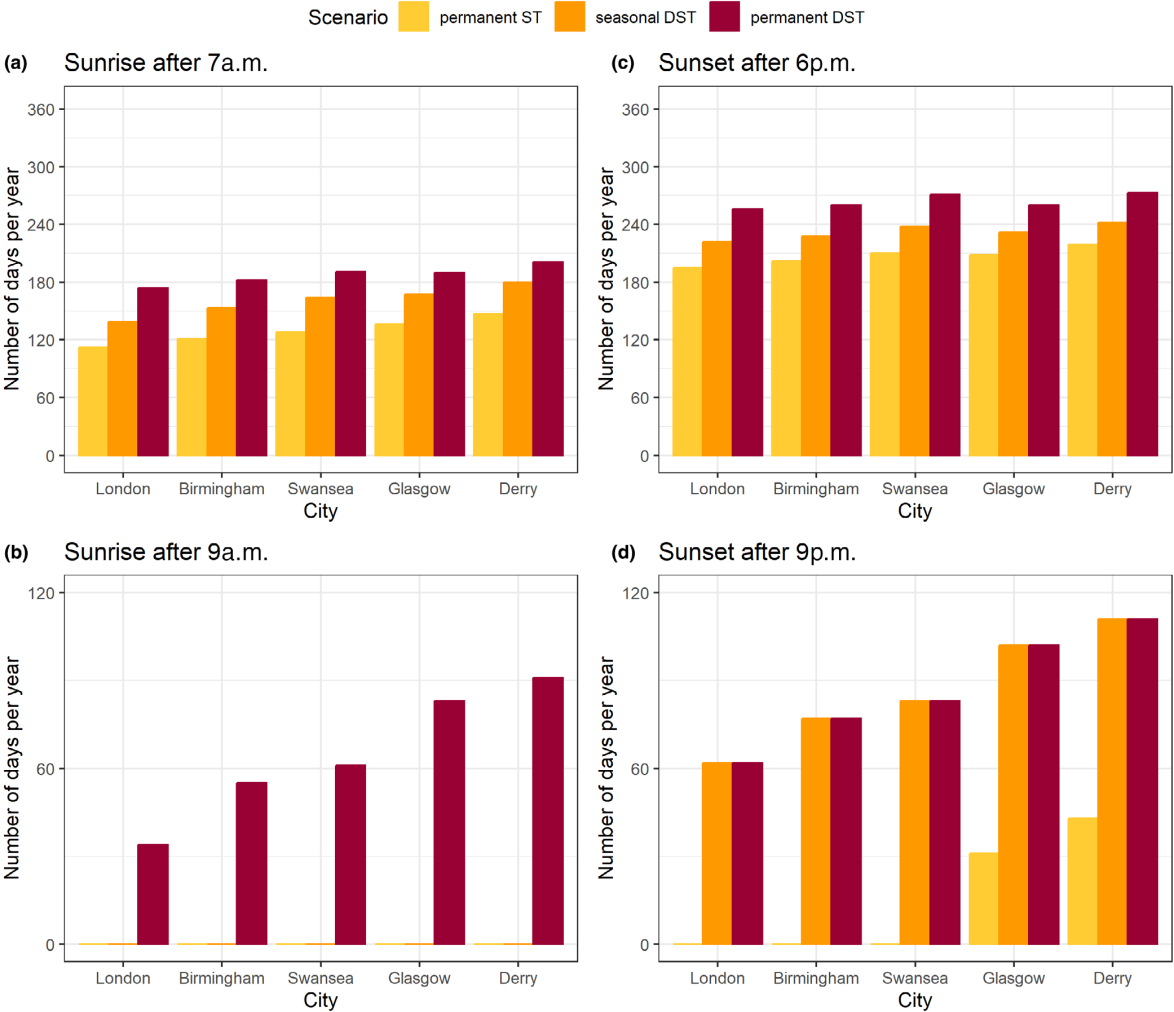
Number of days per year with sunrise after 07:00h  and 09:00h , and sunset after 18:00 hours and 21:00 hours in cities across the UK for the scenarios of permanent Standard Time (pST), seasonal Daylight Saving Time (DST) and permanent DST (pDST). Data are for 2024 (366 days as a leap year). Sunrise and sunset calculations were performed with the R‐package suncalc (doi: 10.32614/CRAN.package.suncalc). Tabular data for these figures are presented in Tables S1 and S2.

British Standard Time follows the Prime Meridian (0° longitude), which passes through the Royal Observatory at Greenwich. This time is also referred to as GMT. Locations along that same longitude will benefit from a close alignment of our clocks and solar time, where solar noon occurs close to noon on our clocks. However, almost the entire UK is located west of the Prime Meridian, and thus experiences not only later solar noon but also later sunrises and sunsets all year round, because the sun rises in the East. In addition, there is a seasonal south–north component influencing sunrise and sunset timing. The further north, the earlier the sun rises and the later it sets during summer (“longer days”). During winter, the opposite applies – later sunrises and earlier sunsets at more northern locations (“shorter days”). Because of the diagonal orientation of the UK, going north usually also means going west (Figure 1), so the year‐round east–west effect on sunrise and sunset is coupled with the seasonal south–north effect. Locations in the north of the UK are thus particularly disadvantaged in winter when it comes to important morning sunlight opportunity because they are also further west (see differences across rows in Table 1 and across locations on the map in Figure 1). This means late sunrises and less time between sunrise and 09:00 hours at those locations, translating into less opportunity for exposure to morning natural light before work and school (Table 1, as well as Figure 2 indicating number of days with sunrises after 07:00 hours [top left panel] and after 09:00 hours [bottom left panel]).

This unique geographical orientation of the UK is important to consider as we consider the impact of DST. There are three solutions to the debate surrounding DST: Keeping the status quo (twice‐yearly clock changes); a permanent change to either Standard Time; or year‐round DST. In the next section, we will briefly outline each of the three scenarios, and their likely impact on sleep and daily rhythms for the UK population. For more extensive exploration of these possibilities, see Malow (2022) and Rishi et al. (2024).

## 4 KEEPING THE STATUS QUO (TWICE‐YEARLY CLOCK CHANGES)

The current practice of moving our clocks – and thus our daily schedules – forward by 1 hour for 7 months from March to October has been criticised for several reasons. From a sleep and biological rhythms perspective, the switch to DST is detrimental, firstly because of its immediate effects on sleep and circadian rhythms elicited by the abrupt switches, and secondly because of the potential chronic effects of having schedules 1 hour earlier in relation to solar time for over half a year. The acute effects are most detrimental in Spring when the nation loses an hour of their night leading to acute sleep loss. Numerous transient adverse effects on health, performance, productivity and safety for both paediatric and adult populations have been linked to the Spring DST change (Hurst et al., 2024; Medina et al., 2015; Orsini et al., 2024; Osborne‐Christenson, 2022; Robb & Barnes, 2018; Zhang et al., 2020). Effects on mortality and traffic safety are inconclusive (Carey & Sarma, 2017; Klerman et al., 2024; Levy et al., 2022; Poteser & Moshammer, 2020; Zhao et al., 2024). While the individual risk may be small for most people, because this physiological disruption is imposed on millions of people at the same time, there appears to be an adverse effect on public health, productivity and safety at the population level.

The long‐term physiological effects of observing DST for 7 months of the year are more challenging to determine because they are difficult to separate out from seasonal effects. However, some research in locales with unique DST regulations enabling such separation indicated negative long‐term impacts on circadian rhythms, well‐being and performance (Borisenkov, 2011; Giuntella & Mazzonna, 2019). Basic circadian physiology suggests that *most* negative consequences for our sleep and body clocks from seasonal DST would arise less from the summer months where daylight is plentiful and sunrises are early, but from the first and last few months of DST in spring and autumn when sunrises are much later. During these months, starting the day 1 hr earlier as demanded by DST reduces opportunity for important morning sunlight exposure, jeopardising the optimal regulation of our circadian rhythms and sleep.

Furthermore, while sunlight exposure *before* work/school around mid‐summer might not be negatively affected by DST given the early sunrises, the opportunity for light exposure *after* work/school is over‐expanded in the summer months leading to significant sunlight close to bedtime – or even beyond bedtime for children or adults with early schedules (Table 2 or Figure 2, bottom right panel: sunset after 21:00 hours; note that dusk is also extended during summer leading to daylight long after sunset). Significant light exposure close to bedtime makes it difficult to fall asleep, through circadian mechanisms, but also via direct suppression of the sleep‐promoting hormone melatonin and the direct alerting effects of light (Blume et al., 2019; Brown et al., 2022). Hence, the additional DST‐induced delay of the already late sunsets in summer (relative to bedtime) makes it difficult to achieve a full night's sleep in summer, particularly for individuals requiring more sleep, such as children. Taken together, seasonal DST with its twice‐yearly clock changes is likely suboptimal for sleep and circadian physiology, with potential risks for health and productivity.

**TABLE 2:** Overview of sunset times in the scenario of permanent British Standard Time (in 2024) and permanent DST with data sourced from https://www.timeanddate.com/.

		Permanent Standard Time option	Permanent DST option
	City	Sunset	Sunset
		Summer solstice	Summer solstice
		Time (hours)	Time (hours)
Time	London	20:21	21:21
	Birmingham	20:34	21:34
	Swansea	20:37	21:37
	Glasgow	21:06	22:06
	Derry/Londonderry	21:12	22:12

DST, Daylight Saving Time.

## 5 IMPLEMENTING PERMANENT DST

As seductive as permanent “Daylight Saving Time” or “Summer Time” may sound, because the hours of daylight in the UK change across the seasons, we would still have shorter days during winter even under permanent DST. Maintaining DST beyond summer and into winter in the UK would deprive the UK population (especially in the northern and western parts of the country) of natural light during the crucial morning hours when we have the greatest physiological need for it (columns 6–9 in Table 1, and panel 2 in Figure 1).

Abolishing the twice‐yearly clock change and fixing our time zone at permanent DST would mean replacing a time zone that is fitting for the lighting conditions in London with one that is fitting for the lighting conditions further East (e.g. Western Poland). Living in the UK but effectively following Central European Time would mean that most people would have to get up in the morning at least 1 hr in advance of their local solar time. Even though this would be suboptimal in London, the consequences would be even worse further north and further west (Figures 1 and 2; Table 1).

The main argument put forward for adopting permanent DST, other than the semantic appeal of extending “summer time”, is the suggestion that people with regular day jobs and children in full‐time education would have the opportunity to experience more natural light in the evening after work/school, as light is redistributed from before work to after work by getting up an hour earlier relative to our previous schedules. However, compared with seasonal DST, permanent DST would only provide additional daylight after the end of the workday and its purpoted benefits for a few weeks in late Autumn and early Spring. The photoperiod during the winter months is so short that implementing permanent DST would not prevent darkness after work/school in the winter ‐ there are simply not enough daylight hours to “save”. Crucially, though, for every minute of this evening daylight, we would sacrifice the same amount of morning light, and the lack of light in the morning is greatest in the North‐West of the country. Glasgow would not see the sun rise until 09:45h  in the winter if we adopted DST permanently (Table 1, column 9; Figure 1, panel 2, and Figure 2, both left panels).

In the absence of direct epidemiological studies, the long‐term risks of living in the wrong time zone can be extrapolated from studies comparing sleep and health between those who live on the westerly to those on the easterly margin of a time zone. They observe the same clock schedule but experience big differences in solar time. These studies, mainly conducted along the 1‐hour‐wide time zones of the USA, indicate consistently poorer sleep and poorer health the further west a location is in the time zone, that is, the later solar timing is in relation to clock time. These detriments include less sleep, dysregulated circadian rhythms, higher cancer incidence, more fatal traffic accidents, increased rates of obesity, poorer cardiovascular health, increased rates of suicide, and shorter life expectancy (Borisenkov, 2011; Gentry et al., 2022; Giuntella & Mazzonna, 2019; Gu et al., 2017; Reis et al., 2023). These data suggest that the negative circadian and sleep effects outweigh the purported benefits of permanent DST (temporary increase in mood, or increased opportunity for evening exercise).

Thus, whilst the epidemiological data to formally demonstrate the long‐term effects of permanent DST are challenging to obtain, because conditions to test this rarely exist, it is likely that implementation of permanent DST in the UK would represent a risk for our sleep, health and productivity.

## 6 RESTORING PERMANENT STANDARD TIME (GMT)

Restoring permanent Standard Time would mean abolishing the twice‐yearly clock changes entirely, and our clocks would be closely aligned to solar time. It would mean earlier sunsets in the summer, but considering sunset in the UK during midsummer would still be between 20:15h  and 21:15h  (Table 2), and we would have a significant number of days with sunsets after 18:00h  (Figure 2, top right panel), it would still allow us to engage in outdoor after work/school activities. There would likely be additional benefits to health from improved sleep and circadian alignment via increased morning sunlight exposure from autumn to spring (Table 1, columns 2–5; Figure 1, panel 1) and a reduction in number of days with sunsets after 21:00 h (Figure 2, bottom right panel) as described above.

Considering the arguments listed above, the British Sleep Society recommends the restoration of permanent Standard Time in the UK (GMT). From a sleep and circadian health perspective, this option is preferable to twice‐yearly changes to DST, and strongly preferable to permanent DST.

## 7 AVOIDING A TIME ZONE BOUNDARY WITH IRELAND

One last important consideration for the UK is the time zones on both sides of the Irish border. Currently, both the UK and Ireland observe GMT (and DST in the summer). It is imperative that if the UK considers changes to the current time zone arrangements, the discussion and the decision are taken in partnership with the Republic of Ireland to avoid a time zone border across the island.

## 8 CONSENSUS POSITION STATEMENT OF THE BSS

**The British Sleep Society strongly recommends the restoration of permanent Standard Time (GMT) in the UK.**

The position of the British Sleep Society, based on current evidence, is:

1. Abolish the twice‐yearly clock change to prevent the acute adverse effects on sleep, health, performance and safety.
2. To advise against any proposal to impose permanent DST in the UK, which would create significant risks to public health and well‐being that would be mostly born by the already disadvantaged regions outside London and the Home Counties.
3. To strongly recommend the return to permanent Standard Time (GMT) across the UK. This will minimise any potential health risk from sleep and circadian disturbances.

This position statement is supported by the following organisations and networks:

The Irish Sleep Society
British Paediatric Sleep Society
Capella (Sleep Action)
The Sleep Charity
The British Society of Pharmacy Sleep Services
Circadian Mental Health Network.

## AUTHOR CONTRIBUTIONS

**Megan R. Crawford:** Conceptualization; investigation; funding acquisition; writing – original draft; writing – review and editing; visualization; project administration; supervision. **Eva C. Winnebeck:** Writing – original draft; conceptualization; writing – review and editing; visualization. **Malcolm von Schantz:** Writing – original draft; conceptualization; writing – review and editing; visualization. **Maria Gardani:** Writing – original draft; writing – review and editing; conceptualization. **Michelle A. Miller:** Writing – original draft; writing – review and editing; conceptualization. **Victoria Revell:** Writing – original draft; writing – review and editing; conceptualization. **Alanna Hare:** Writing – review and editing; funding acquisition. **Caroline L. Horton:** Writing – review and editing; funding acquisition. **Simon Durrant:** Writing – review and editing; funding acquisition. **Joerg Steier:** Writing – review and editing; funding acquisition; conceptualization.

## FUNDING INFORMATION

The British Sleep Society commissioned the creation of figure 1. No external funding was received for this project.

## CONFLICT OF INTEREST STATEMENT

MC is a consultant for Signifier Medical Technologies and has received research funding from BRUK. EW has received research funding from the Deutsche Forschungsgemeinschaft (DFG, German Research Foundation). MG has received funding from Capella Charity for running training events. MM has received research funding from NIHR, royalties from Oxford University Press for Sleep, Health and Society Textbooks. MvS has received research funding from the MRC, Wellcome Trust and NIH. VR has received research funding from the Ministry of Defence, the Ministry of Justice, UKDRI and NIHR, she is also a scientific advisor for Lumie. AH has received speaker fees from Idorsia Pharmaceuticals, Holland and Barrett, and Fisher and Paykel. These activities have not influenced the submitted work. All other authors report no financial relationships with any organisations that might have an interest in the submitted work in the previous 3 years, and no other relationships or activities that could appear to have influenced the submitted work. All authors are members of the BSS Executive Committee or BSS executive committee co‐opted subgroups. The British Sleep Society paid for the production of the image for the manuscript (payment received by Oliver Burdekin at burdGis).

## Supporting information


**TABLE S1.** Number of days with sunrise after 07:00h and 09:00h in cities across the UK for the scenarios of permanent Standard Time, seasonal DST and permanent DST.
Data are for 2024 (366 days as it is a leap year). Sunrise calculations were performed with the R‐package suncalc (doi: 10.32614/CRAN.package.suncalc).
**TABLE S2.** Number of days per year with sunset after 18:00h and 21:00h in cities across the UK for the scenarios of permanent Standard Time, seasonal DST and permanent DST.
Data are for 2024 (366 days as it is a leap year). Sunset calculations were performed with the R‐package suncalc (doi: 10.32614/CRAN.package.suncalc).

## Data Availability

Data sharing not applicable to this article as no datasets were generated or analysed during the current study.
