# Supplementary material for: The British Sleep Society position statement on Daylight Saving Time in the UK
Source: J Sleep Res. 2024 Oct 23;34(3):e14352. doi: 10.1111/jsr.14352 (PMC12069735; doi:10.1111/jsr.14352)
Supplement: Supplementary file 1 — TABLE S1. Number of days with sunrise after 07:00h and 09:00h in cities across the UK for the scenarios of permanent Standard Time, seasonal DST and permanent DST. Data are for 2024 (366 days as it is a leap year). Sunrise calculations were performed with the R‐package suncalc (doi: 10.32614/CRAN.package.suncalc). TABLE S2. Number of days per year with sunset after 18:00h and 21:00h in cities across the UK for the scenarios of permanent Standard Time, seasonal DST and permanent DST. Data are for 2024 (366 days as it is a leap year). Sunset calculations were performed with the R‐package suncalc (doi: 10.32614/CRAN.package.suncalc). [file JSR-34-e14352-s001.docx]

#

Table S1:Number of days with sunrise after 7am and 9am in cities across the UK for on the scenarios of permanent Standard Time, seasonal DST and permanent DST. Data are for the year 2024 (366 days as a leap year). Sunrise calculations were performed with the R-package suncalc (doi: 10.32614/CRAN.package.suncalc).

|  | Number of days with sunrise | | | | | |
| --- | --- | --- | --- | --- | --- | --- |
| City | after 7am on permanent ST | after 7am with seasonal DST | after 7am with permanent DST | after 9am on permanent ST | after 9am with seasonal DST | after 9am with permanent DST |
| London | 112 | 139 | 174 | 0 | 0 | 34 |
| Birmingham | 121 | 153 | 182 | 0 | 0 | 55 |
| Swansea | 128 | 164 | 191 | 0 | 0 | 61 |
| Glasgow | 136 | 167 | 190 | 0 | 0 | 83 |
| Derry/Londonderry | 147 | 180 | 201 | 0 | 0 | 91 |

Table S2: Number of days per year with sunset after 6pm and 9pm in cities across the UK for on the scenarios of permanent Standard Time, seasonal DST and permanent DST. Data are for the year 2024 (366 days as a leap year). Sunset calculations were performed with the R-package suncalc (doi: 10.32614/CRAN.package.suncalc).

|  | Number of days with sunset | | | | | |
| --- | --- | --- | --- | --- | --- | --- |
| City | after 6pm on permanent ST | after 6pm with seasonal DST | after 6pm with permanent DST | after 9pm on permanent ST | after 9pm with seasonal DST | after 9pm with permanent DST |
| London | 195 | 222 | 256 | 0 | 62 | 62 |
| Birmingham | 202 | 228 | 260 | 0 | 77 | 77 |
| Swansea | 210 | 238 | 271 | 0 | 83 | 83 |
| Glasgow | 208 | 232 | 260 | 31 | 102 | 102 |
| Derry/Londonderry | 219 | 242 | 273 | 43 | 111 | 111 |
